# Gel-Synthesized Zirconium Diboride (ZrB**_2_**) Powders for All-Solid-State Symmetric and Zinc-Ion Hybrid Supercapacitor

**DOI:** 10.1021/acsomega.5c12290

**Published:** 2026-06-20

**Authors:** S. Altun, E. Karaca, M. Tuncer, E. Erdem, H. Göçmez

**Affiliations:** † Department of Materials Science and Engineering, Graduated Education Institute, Kütahya Dumlupınar University, Kütahya 43100, Turkiye; ‡ Chemistry Department, Faculty of Science, Hacettepe University, Beytepe, Ankara 06800, Turkiye; § Department of Metallurgy and Materials Engineering, Kütahya Dumlupınar University, Kütahya 43100, Turkiye; ∥ Department of Material Science and Nano-Engineering, Sabancı University, Orhanli, Istanbul 34956 Turkiye

## Abstract

This study reports the synthesis and electrochemical evaluation of gel-synthesized ZrB_2_ for all-solid-state symmetric supercapacitors. The prepared ZrB_2_ powders were characterized using XRD, SEM, FTIR, and XPS analyses. Electrochemical performance was tested using cyclic voltammetry (CV) in 1 M Na_2_SO_4_ electrolyte in both positive and negative voltage ranges at a wide range of scan rates from 5 to 500 mV/s. Specific capacitances ranged from 18 to 55 F/g in the positive region and 19–73 F/g in the negative region. Dunn's method analysis revealed that pseudocapacitive contributions dominated at low scan rates (80%), while electric double-layer capacitance (EDLC) contributions dominated at high scan rates (70%), indicating hybrid energy storage behavior. A solid-state symmetric supercapacitor cell was assembled using ZrB_2_ electrodes with PVA/Na_2_SO_4_ gel electrolyte, exhibiting a low internal resistance of 2.79 Ω·cm^2^ and an operational voltage of 1.4 V with rectangular CV profiles. Galvanostatic charge–discharge (GCD) tests showed an energy density of 5.0 Wh/kg and a power density of 5600 W/kg at 8 mA cm^–2^. The cell exhibited good cyclic stability of 90.7% after 5000 cycles. Additionally, a gel-derived ZrB_2_ electrode was successfully applied in a solid-state zinc-ion hybrid supercapacitor configuration, demonstrating a high specific capacity of 230 mAh/g at 0.9 C and excellent reversibility with 87% Coulombic efficiency. These results confirm the versatility of gel-synthesized ZrB_2_ for both symmetric and hybrid energy storage systems, making it a promising electrode material for multifunctional solid-state devices.

## Graphical abstract

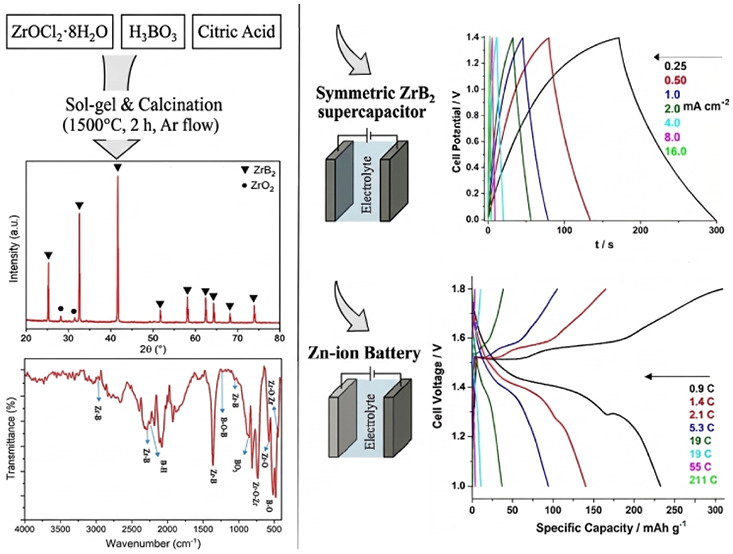

## 1 Introduction

Due to their ability to harvest energy from various sources and efficiently manage energy conversion to the required forms, energy systems play a vital role in applications in various sectors such as public utilities, industry, and transportation.^1–3^ To date, electricity generation has primarily relied on fossil fuels because of their efficiency and reliability. The reduction in the use of fossil fuels and the integration of renewable energy systems into the grid to meet increasing demand offer more economical solutions, particularly because of the continuously rising prices of fossil fuels. Furthermore, nature-friendly characteristics can easily be improved, making energy storage devices increasingly important. Because of the rapid emergence of various renewable energy sources, the need for energy storage systems that can work harmoniously with these sources has increased.^4–6^ Although rechargeable batteries are promising power sources for sustainable energy sources such as wind or solar energy and electric vehicles, their energy storage mechanisms are limited to cathode diffusion in the crystal structure. These mechanisms significantly reduce the charge–discharge rate of batteries. Supercapacitors, which include types such as electrochemical double-layer capacitors (EDLCs) and pseudocapacitors, allow significantly higher power densities compared to batteries because their charge storage system is based on surface reactions on the electrode materials without involving ion diffusion within the bulk material. Supercapacitors, also called electrochemical energy storage systems, have attracted the attention of scientists with their capabilities of high cycle life, high specific power, and fast charge–discharge rate. The electrode material selection is critical for supercapacitor performance.^7–10^ Although the low specific energy of supercapacitors is a disadvantage, recent research has aimed to increase the specific energy values by using advanced electrode materials and developing new supercapacitor systems.

Carbon-based electrodes, such as activated carbon, carbon nanotubes, graphene, biomass-derived carbons, etc., are the most common electrode materials in supercapacitors due to their abundance, low cost, nontoxicity, high surface area, controllable porosity, and excellent electrical conductivity, enabling efficient charge storage and fast cycling.^11,12^ It has been determined that using nanostructured carbons such as carbon dots and graphene quantum dots as electrodes optimizes the physical and chemical properties of the electrode and accordingly increases the energy density and lifespan of the supercapacitors.^13–15^ In addition to carbon-based electrodes, metal oxides and metal hydroxides are suitable for supercapacitors due to their high theoretical capacitance, high redox activity, and good cycling properties.^16–20^ Conducting polymer electrodes, such as polyaniline, polypyrrole, and polythiophene, are used in various supercapacitor applications due to their high pseudocapacitance and flexible properties.^18,21,22^ In recent years, metal borides/transition-metal borides, which are classified as ultrahigh-temperature materials, have attracted great interest as supercapacitor electrode materials.^23–27^ Thanks to their excellent electrical conductivity, chemical stability, and usability in extreme environments, they have emerged as powerful electrode materials for various applications. Ni-cobalt borides, the most commonly used metal borides, show very high specific capacitance (2415 F/g) and high energy density (74.3 Wh/kg), primarily when produced at the nanoscale or combined with metaborates or carbon derivatives. The electrode performance of borides such as NiB, SiB, CoB, B_4_C, and HfB_2_ has been investigated, and promising results have been reported.^28–33^

To the best of our knowledge, studies on the electrochemical performance of ZrB_2_ mainly focus on ZrB_2_ powder and ZrB_2_–SiC and ZrB_2_–ZrC composite powders. According to these studies, ZrB_2_ powders and their composites were synthesized via a mechanical activation-assisted direct synthesis route and the energy and power density values of pure ZrB_2_ powder were reported as 4.2–8.8 Wh/kg and 150–118 W/kg, respectively.^34,35^ The energy and power density values of ZrB_2_–SiC composite powders were reported as 0.4 Wh/kg and 75 W/kg, respectively, and the results of energy and power density of ZrB_2_–ZrC composite powders were reported as 5.8 Wh/kg and 158 W/kg, respectively.^34,35^ Electrochemical performance research and results development are essential for all metal borides, especially for ZrB_2_.

Recently, zinc-ion batteries have emerged as a promising alternative to lithium-ion batteries for large-scale energy storage applications due to their inherent safety, low cost, and environmental benignity.^36,37^ The aqueous electrolyte systems in zinc-ion batteries eliminate fire hazards and enable operation under ambient conditions. However, zinc-ion batteries face significant challenges, including limited cathode material options, zinc dendrite formation, and capacity degradation during cycling. Metal borides, particularly transition-metal borides such as TiB_2_ ^38^ and ZrB_2_, offer unique advantages for zinc-ion battery cathodes due to their high electronic conductivity, structural stability, and ability to accommodate zinc-ion intercalation/deintercalation. The robust crystal structure of metal borides can effectively suppress volume expansion during cycling, while their metallic conductivity ensures efficient electron transport, making them attractive candidates for high-performance zinc-ion battery cathodes. In a related study, the addition of small amounts of TiB_2_ (1–5 wt %) to MnO_2_ cathodes was found to increase the capacity from 150 mAh g^–1^ to 220 mAh g^–1^.^38^ The TiB_2_ additive facilitates lithium insertion while suppressing proton insertion.

In contrast to these mechanically activated systems, this work (i) synthesizes ZrB_2_ predominantly (with minor ZrO_2_) via a citrate-gel route that affords uniform particle morphology,^39^ (ii) integrates the gel-derived ZrB_2_ into an all-solid-state symmetric supercapacitor with a PVA/Na_2_SO_4_ gel electrolyte, and (iii) incorporates a solid-state zinc-ion hybrid supercapacitor employing a PVA/2 M ZnSO_4_–0.2 M MnSO_4_ gel electrolyte. The prepared ZrB_2_ powder was analyzed using XRD, SEM, XPS, and FTIR to investigate the structural properties and surface chemistry. In order to evaluate the capacitive performance, the ZrB_2_-based electrode was investigated by cyclic voltammetry (CV) in 1 M Na_2_SO_4_ solution. The CV tests were performed at various scan rates ranging from 5 to 500 mV s^–1^ to evaluate both capacitive and diffusive contributions to total charge storage. The measurements were performed in both positive (0 to 0.8 V) and negative (0 to −0.8 V) potential regions to identify the optimum operating voltage range in the 1 M Na_2_SO_4_ solution. A symmetric supercapacitor cell was constructed with ZrB_2_-based electrodes as positive and negative sides to assess its practical performance. A cost-effective and environmentally friendly PVA/Na_2_SO_4_ gel electrolyte was used in the cell due to its mechanical properties and high ionic conductivity.

## 2 Experimental Section

### 2.1 Synthesis of ZrB_2_ Powders

The synthesis procedure of zirconium diboride powders via the citric acid gel method was detailed in our previously published paper,^40^ as seen in Scheme 1. Briefly, aqueous solutions of zirconium oxychloride (ZrOCl_2_·8H_2_O, Fluka, >99%), citric acid (C_6_H_8_O_7_, VMR Chemicals, 100%), and boric acid (H_3_BO_3_, Eti Mining) were separately dissolved in deionized (DI) water to obtain transparent solutions, which were then mixed to form a homogeneous solution. This solution was stirred for 24 h at room temperature and subsequently heated at 90 °C until xerogel formation. The resulting gel was heat-treated at 1500 °C for 2 h, yielding ZrB_2_ powders. XRD analysis was performed to determine the phase content in the powders by using Cu Kα radiation (45 kV, 35 mA, λ = 1.54 Å) with a scanning range of 5–80°. The structure and morphology of synthesized powders were analyzed by field emission scanning electron microscopy (SEM – FEI/Nova NANOSEM 650) with EDX (EDAX). X-ray photoelectron spectroscopy (XPS) analysis was performed using an AXIS HSI 165 spectrometer with a monochromated Al Kα X-ray source (photon energy: 1486.71 eV).

**Scheme 1:**
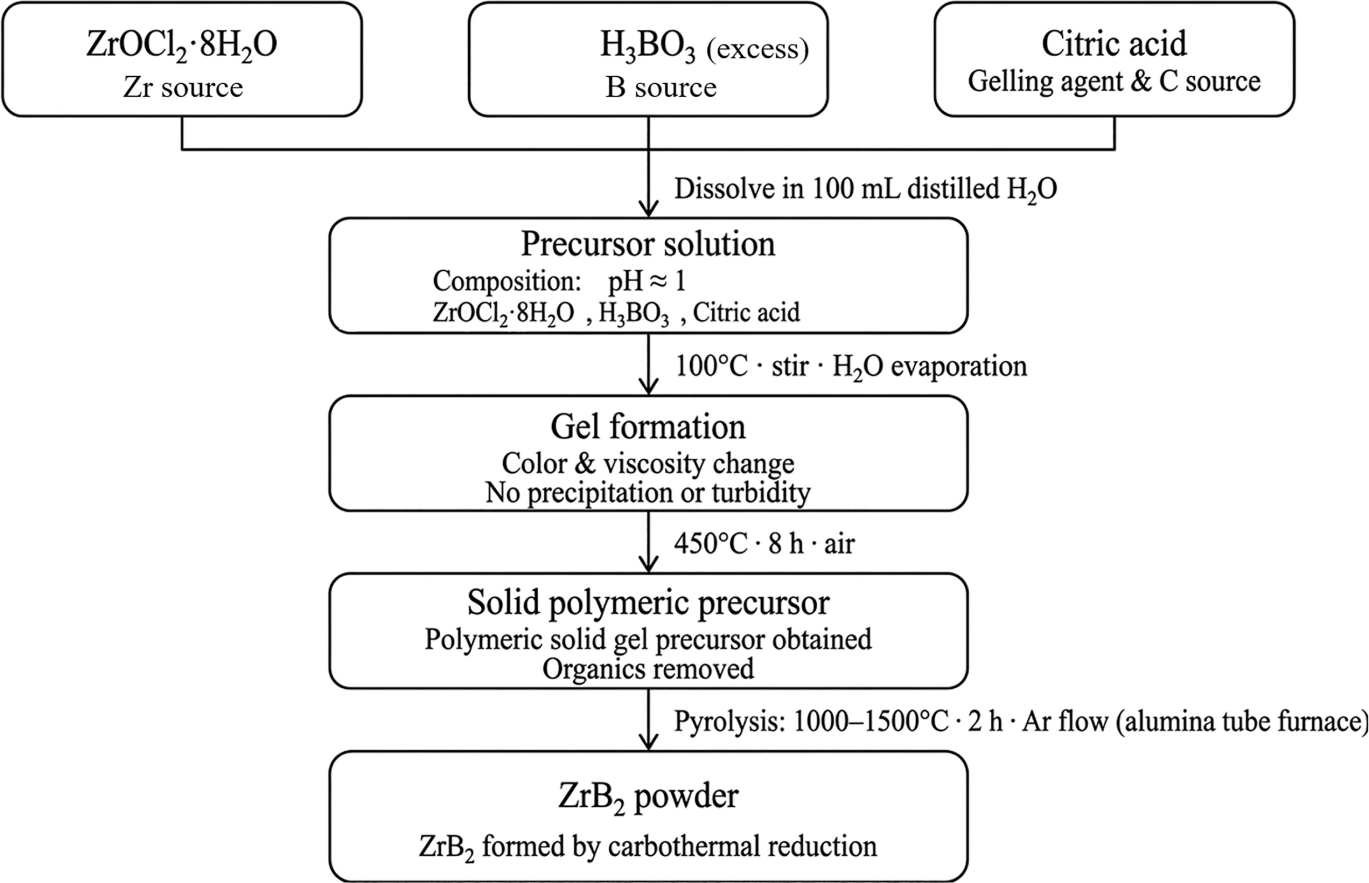
Synthesis Pathway of ZrB_2_ Powders by Citrate Gel Process

### 2.2 Electrochemical Tests of ZrB_2_ Electrodes

The ZrB_2_ electrode was coated on nickel foam using a drop-casting and drying method with 80% active material, 10% polyvinylidene fluoride/*N*-methyl-2-pyrrolidone, and 10% carbon black. After drying at 80 °C in a vacuum oven for 12 h, the electrode was pressed from 1.5 mm to 0.2 mm. The mass loading was ∼1.0 mg/cm^2^. Ag/Ag^+^ was used as the reference electrode, and a graphite rod was used as the counter electrode. Electrochemical experiments were conducted using a Biologic SP240 potentiostat/galvanostat. The electrode was tested using electrochemical impedance spectroscopy (EIS) and CV technique in a three-electrode cell in 1 M Na_2_SO_4_ solution in both positive and negative regions, and the related specific capacitance values were calculated.

In the two-cell configuration of a solid-state supercapacitor, the prepared electrodes were used as both the anode and the cathode to construct a solid-state symmetric supercapacitor. A gel electrolyte was prepared by mixing 2 g poly­(vinyl alcohol) (PVA) and 2 g Na_2_SO_4_ in 20 mL of water and heating at 80 °C. The gel electrolyte was applied to both sides of a microfiber filter paper separator (Whatman), and the electrodes were placed to construct a two-electrode split test cell. EIS, CV, and GCD tests were performed. Energy and power density, Coulombic efficiency, and cycle life (after 1000 cycles) were determined. Additionally, a solid-state zinc-ion battery was assembled using a ZrB_2_ electrode as the cathode and zinc foil as the anode. A gel electrolyte was prepared by mixing 2 g PVA and 2 M ZnSO_4_/0.2 M MnSO_4_ in 20 mL of water and heating at 80 °C. A two-electrode split test cell was also assembled by coating both faces of a Whatman microfiber filter paper separator with the gel electrolyte and positioning the electrodes on either side. EIS, CV, and GCD tests were performed to determine the internal resistance, capacity, and rate capability.

## 3 Results and Discussion

### 3.1 Microstructural Properties

Figure 1a presents the XRD pattern of the powders sintered at 1500 °C. The diffraction peaks with hkl indices at 41.8° (011), 32.8° (010), 25.1° (001), 62.6° (012), 58.2° (110), 74.2° (021), 64.5° (111), 51.9° (002), and 68.3° (020) exhibit relatively strong intensities corresponding to ZrB_2_ (PDF: 98-061-5765), while minor amounts of ZrO_2_ are also detected at 28.2° (11-1) and 31.6° (111) (PDF: 98-009-4887). ZrO_2_ formation is attributed to partial oxidation during processing. XPS analysis of the gel-synthesized powders was performed (Figure 1b) to identify the surface chemical states and confirm the presence of both boride and oxide species. In the deconvoluted Zr 3d spectrum, the peaks at 178.3, 180.9, 182.3, 184.6, 187.5, and 190.5 eV were attributed to Zr 3d_3/2_ and Zr 3d_5/2_ of ZrB_2_, Zr 3d_3/2_ and Zr 3d_5/2_ of ZrO_2_, B 1s of ZrB_2_, and B 1s of B_2_O_3_ (from residual boron oxide species).^41,42^ XPS analysis verified that ZrB_2_ constitutes the predominant phase with a thin surface oxide layer (ZrO_2_/B_2_O_3_), indicating the presence of a hybrid capacitive response observed electrochemically. ZrB_2_ primarily facilitates electric double-layer capacitance, but the thin oxide layer offers additional faradaic sites, hence enabling pseudocapacitive processes.

**Figure 1:**
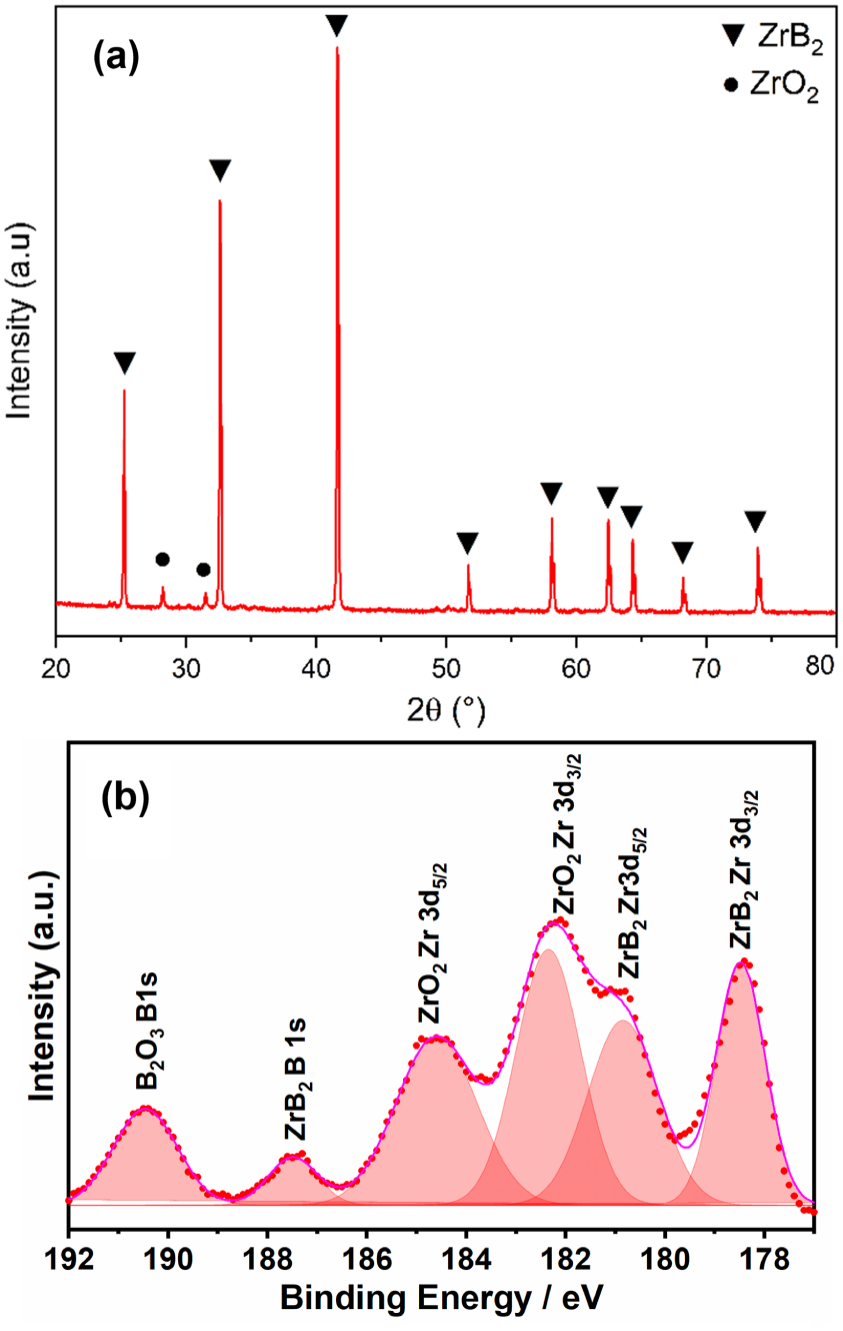
(a) XRD pattern and (b) deconvoluted Zr 3d and B 1s spectra for gel-synthesized powders at 1500 °C.

The SEM micrograph of the ZrB_2_ powder is presented in Figure 2. The ZrB_2_ particles exhibit hexagonal-like morphology, with sizes ranging from the submicrometer to the micrometer scale. Because of the high heat-treatment temperature, agglomeration occurred at the contact points between ZrB_2_ particles. In the EDX spectra, Zr and B peaks confirm the formation of ZrB_2_, with minor peaks arising from the Pt/Pd layer deposited to prevent charging effects.

**Figure 2:**
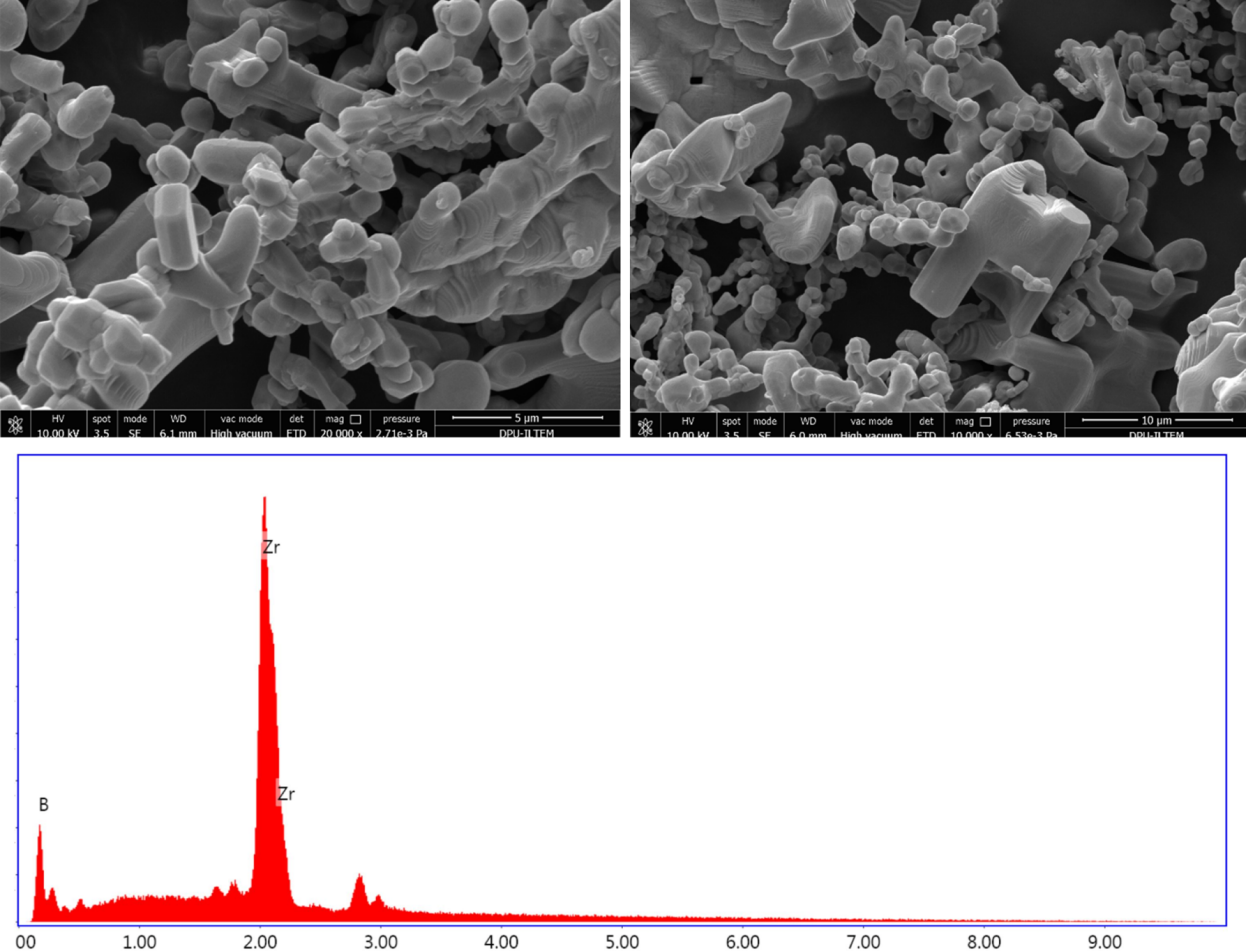
SEM images and EDX spectra of different magnifications of gel-synthesized powders at 1500 °C.

Figure 3 displays the FT-IR spectrum of the gel-synthesized powders at 1500 °C. According to the literature,^43,44^ the absorption bands of 1054 cm^–1^, 1361 cm^–1^, 2283 cm^–1^, and 2949 cm^–1^ are attributed to stretching vibrations of Zr–B bonds. Consistent with the XRD results, Zr–O–Zr bonds were observed at 441 and 728 cm^–1^,^45,46^ while the Zr–O bond was detected at 571 cm^–1^.^47^ These oxygen-related bonds may be attributed to an insufficient amount of citric acid used as a carbon source during synthesis, which may not have effectively eliminated oxygen from the structure. As a result, excess oxygen inhibits the complete reaction between boron and zirconia, leading to the appearance of B–H,^48^ B–O, B–O–B,^49^ and BO_3_ ^50^ vibrations at 2248 cm^–1^ and 513 cm^–1^, 1226 cm^–1^ and 845 cm^–1^, respectively.

**Figure 3:**
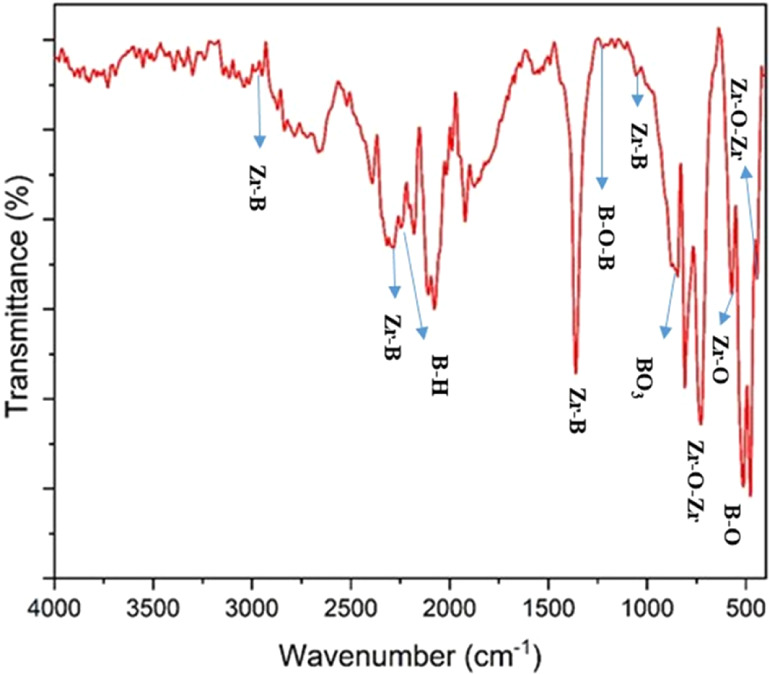
FTIR spectrum of gel-synthesized powders at 1500 °C.

### 3.2 Electrochemical Characterization

#### 3.2.1 Three-Electrode System

The electrical properties of the ZrB_2_-coated nickel foam electrode were investigated using EIS analysis in a three-electrode cell with 1.0 M Na_2_SO_4_ electrolyte. Nyquist and Bode curves were presented in Figure 4. The EIS plot was fitted using the equivalent circuit of R_s_(C_dl_(R_ct_W))­C_pc_. Then, the fitted parameters were found to be 1.01 Ω cm^2^, 0.12 mF cm^–2^, 0.5 Ω cm^2^, 0.03 Ωs^–1/2^ cm^–2^, and 0.015 F cm^–2^, respectively. The near-vertical line in the Nyquist curve at low frequencies and the gradual increase in phase angle in the Bode plot indicate that the coating has low resistance and high capacitive properties. The relaxation time constant (τ = 1/f_0_) was determined from the Bode plot using the characteristic frequency (f_0_), defined as the frequency at which the phase angle reaches −45°.^51^ The relaxation time constant (τ) was estimated as ∼0.6 s, indicating relatively fast charge transfer kinetics and efficient ion transport, consistent with the hybrid capacitive behavior of the electrode.

**Figure 4:**
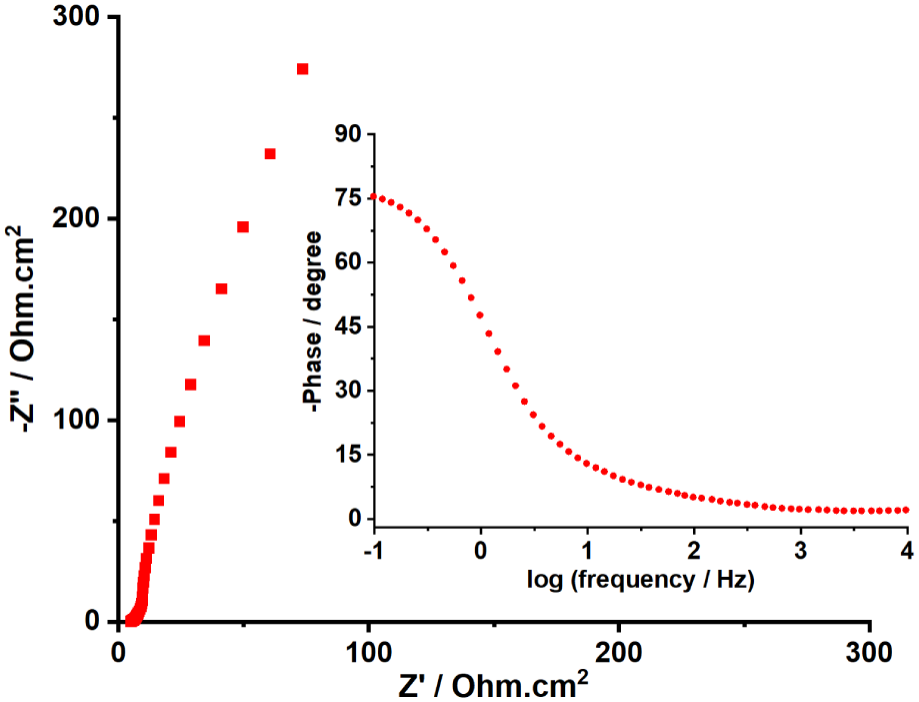
EIS curves for the ZrB_2_ electrode.

The capacitive behaviors of the ZrB_2_ electrode were investigated in a three-electrode cell system using the CV technique in 1.0 M Na_2_SO_4_ electrolyte at 500 to 5 mV s^–1^ in both the positive region (between 0 and 0.8 V) and the negative region (between 0 and −0.8 V) (Figure 5a). Rectangular-type supercapacitor behavior was observed in both regions. In the positive region, the specific capacitances at scan rates of 500, 200, 100, 50, 20, 10, and 5 mV s^–1^ were found as 18, 26, 30, 32, 40, 46, and 55 F g^–1^, respectively, while in the negative region, they were 19, 25, 33, 39, 45, 53, and 73 F g^–1^. A synergistic effect is observed in the ZrB_2_ composite due to both diffusion and pseudocapacitive contributions. The energy storage mechanism occurs through the following reactions:^52^

1 $${\mathrm{ZrO}}_{2}+{\mathrm{Na}}^{+}+e^{-}↔{\mathrm{ZrO}}_{2}-\mathrm{Na}$$

2 $${\mathrm{ZrB}}_{2}+{\mathrm{Na}}^{+}+e^{-}↔{\mathrm{ZrB}}_{2}-\mathrm{Na}$$

**Figure 5:**
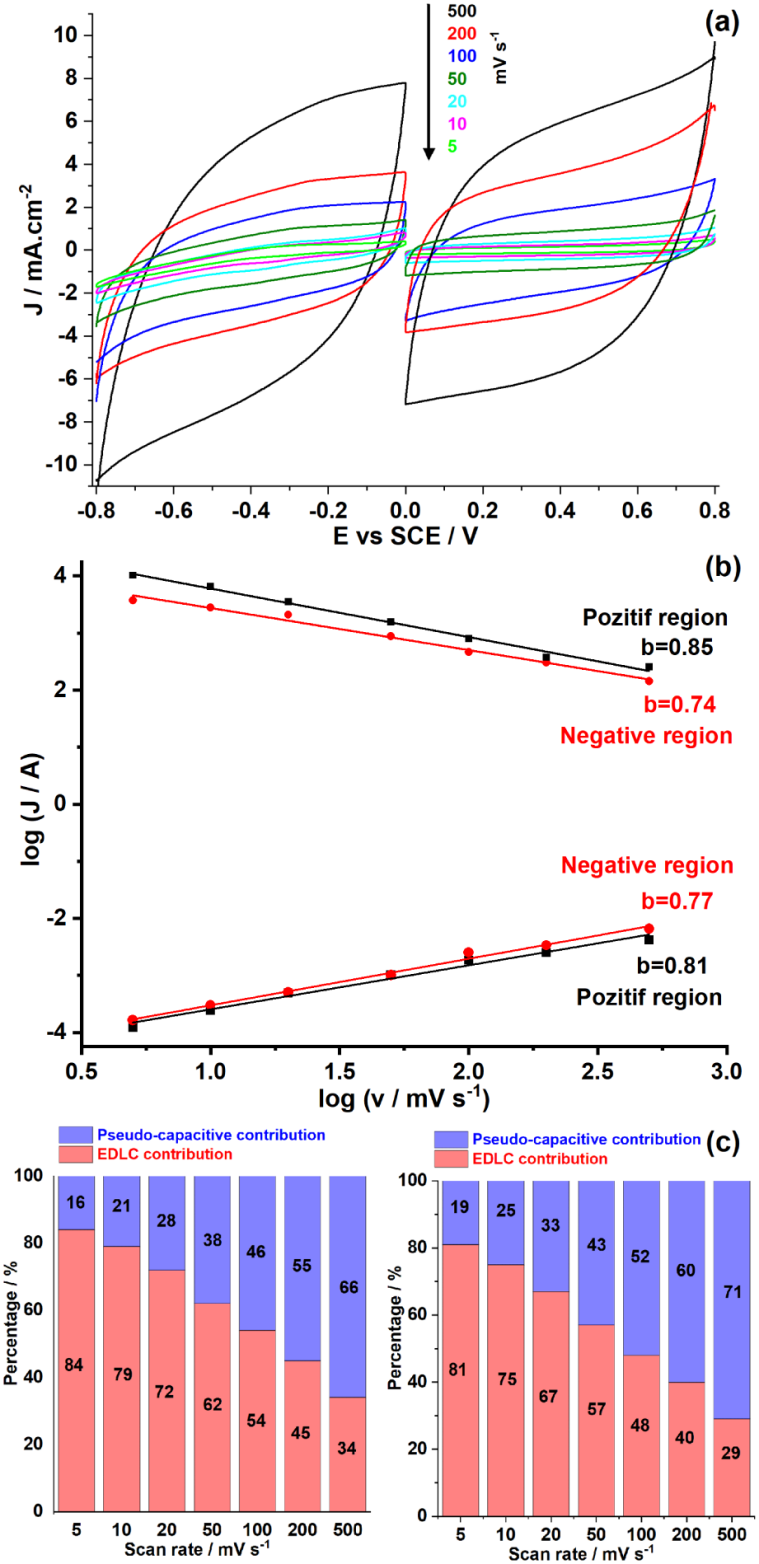
(a) Cyclic voltammograms of the ZrB_2_ electrode recorded at scan rates from 5 to 500 mV s^–1^ in both positive and negative potential windows, (b) log (J/A) vs log (*v*, mV s^–1^) for determining the *b*-values, and (c) the corresponding pseudocapacitive and EDLC contributions.

To evaluate the charge storage kinetics of the ZrB_2_ electrode, the relationship between peak current (*i_p_*) and scan rate (ν) was analyzed. The total capacitance consisted of a capacitive process (fast kinetics) and a diffusion-limited process (slow kinetics). The mechanism was analyzed using the following equations: *i*_*p*_ = *a*·ν*b,* log (*i*_*p*_) = log (*a*) + *b*·log (ν), where *i_p_* is the charge/discharge peak current, ν is the scan rate, and *a* and *b* are adjustable parameters.^53^ A *b*-value of 1.0 indicates a capacitive process, while *b* = 0.5 represents a diffusion-limited process. As shown in Figure 5b, the calculated *b*-values for the anodic and cathodic peaks changed from 0.74 and 0.85, respectively, suggesting hybrid kinetic behavior dominated by surface-induced processes. To further analyze the electrochemical kinetics and quantify the contribution of different charge storage mechanisms, Dunn’s method was employed based on the relationship proposed by Conway et al.^54–56^ The total current at a fixed potential can be expressed as the sum of surface-controlled capacitive effects (*k*_1_·ν) and diffusion-controlled intercalation processes (*k*_2_·ν^1/2^): *i*(*V*) = *k*_1_·ν + *k*_2_·ν^1/2^. This equation can be rearranged to a linear form to determine the constants *k*_1_ and *k*_2_: *i*(*V*)/ν^1/2^ = *k*_1_·ν^1/2^ + *k*_2_. By plotting *i*(*V*)/ν^1/2^ vs ν^1/2^ at each potential, *k*_1_ (the slope) and *k*_2_ (the y-intercept) were calculated.^57,58^ For instance, at a potential of 0.4 V (vs SCE) and a scan rate of 5 mV s^–1^, the values of *k*_1_ and *k*_2_ were determined to be 0.0078 and 0.050, respectively. The resulting capacitive and diffusive contributions across different scan rates are presented as a bar graph in Figure 5c. It was determined that the surface-controlled capacitive contribution increases with the scan rate in both the positive and negative regions.

At low scan rates, the pseudocapacitive contribution is 80%, while at high scan rates, the electric double-layer capacitance (EDLC) contribution is 70%. These results indicate that the electrode exhibits a diffusion-controlled energy storage mechanism at low scan rates and transitions to a surface-controlled mechanism at high scan rates. Ultimately, this demonstrates that the synthesized ZrB_2_ electrode is a hybrid energy storage material.

#### 3.2.2 Two-Electrode System

##### 3.2.2.1 Supercapacitor Studies

A solid-state symmetric supercapacitor cell was prepared in a sandwich configuration, consisting of two identical ZrB_2_ electrodes-coated nickel foam electrodes (each with a mass loading of ∼1.0 mg cm^–2^) placed on either side of a poly­(vinyl alcohol)/sodium sulfate (PVA/Na_2_SO_4_) gel electrolyte-impregnated microfiber separator. Prior to assembly, the electrodes were compressed to ensure close contact and a uniform thickness. The cell was tested using EIS, CV, and GCD techniques (Figure 6) in a two-electrode cell system. The cell exhibited a low internal resistance of 2.79 Ω·cm^2^ from R_s_(C_dl_(R_ct_W))­C_pc_ equivalent circuit (Figure 6a). From the Bode plot, the relaxation time constant (τ) was estimated to be ∼0.13 s, indicating fast charge transfer kinetics and efficient ion transport within the electrode/electrolyte system. In cyclic voltammograms of the cell (Figure 6b), the operational voltage window was determined to be 1.4 V, and the cell maintained the rectangular shape over a wide range of 5–500 mV s^–1^, indicating ideal capacitive behavior. GCD curves were recorded at current densities of 0.25–16.0 mA cm^–2^, as seen in Figure 6c. An energy density of 5.0 Wh kg^–1^ and a power density of 5600 W kg^–1^ were obtained at 8 mA cm^–2^ with a coulombic efficiency of 63% (Table 1). After 5000 cycles (Figure 6 (d)), 90.7% cycle stability was achieved at 8 mA cm^–2^, demonstrating excellent long-term cycling stability (Figure 6c).

**Table 1:** Capacitive Properties of ZrB_2_-Based Solid-State Symmetric Supercapacitor Cell Obtained from GCD Curves (Figure 6c)

J/mA cm^–2^	E/Wh kg^–1^	P/W kg^–1^	Coulombic Efficiency/%
0.25	6.5	175	52
0.50	6.0	350	55
1.0	5.9	700	62
2.0	5.7	1400	76
4.0	5.6	2800	69
8.0	5.0	5600	63
16.0	4.4	11200	83

**Figure 6:**
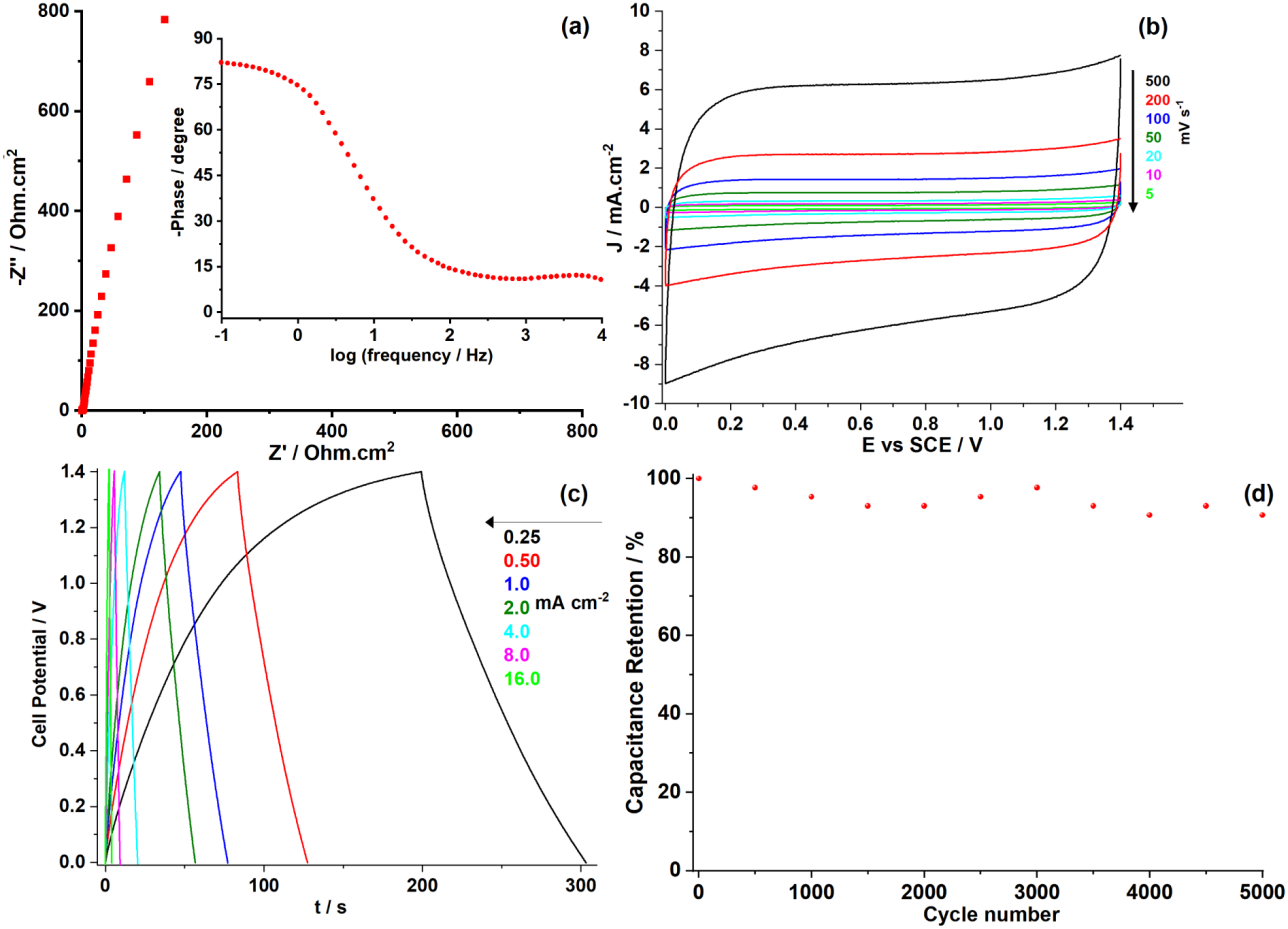
(a) EIS, (b) CV, (c) GCD, and (d) cycle life tests for the solid-state symmetric supercapacitor cell and PVA//Na_2_SO_4_:H_2_SO_4_ electrolyte.

##### 3.2.2.2 Zinc-Ion Battery Studies

A solid-state zinc-ion battery constructed with a ZrB_2_ electrode (∼1.0 mg cm^–2^) as the cathode and zinc foil as the anode was tested in PVA/2 M ZnSO_4_/0.2 M MnSO_4_ in 20 mL of water and heating at 80 °C. Before testing, the cell was charged at 1.8 V for 1 h to electrodeposit MnO_2_ (∼4.5 μg cm^–2^). CV curves of zinc metal and ZrB_2_/MnO_2_ electrode were recorded in a three-electrode cell with zinc metal as both counter and reference electrodes (Figure 7a). When the cathode was tested between 1.0 and 1.8 V, the anode was tested between −0.2 and 1.0 V. The Zn plating/stripping process is highly stable, as shown by the nearly overlapping CV curves.^59^ On the cathode, two reversible redox peaks were observed due to Mn^4+^/Mn^3+^ and Mn^3+^/Mn^2+^. The cathode CV profiles remain unchanged after the first cycle, confirming good reversibility. When EIS of the cell was recorded (Figure 7b), the internal resistance was found to be 13.5 Ω·cm^2^. The relatively low internal resistance indicates efficient ion transport and good interfacial contact within the solid-state zinc-ion cell. The relaxation time constant from the Bode curve was estimated to be approximately ∼100 s, indicating relatively slow ion diffusion and charge-transfer kinetics in the zinc-ion battery system compared with the supercapacitor configuration. This result is consistent with the diffusion-controlled faradaic storage mechanism of Zn-ion systems. For the cell, ZrB_2_ provides high conductivity and mechanical strength,^38^ while ZrO_2_ and B_2_O_3_ act as protective and binding agents. The cell was tested using GCD at various C-rates between 0.9 and 211 C (Figure 7c), with higher C-rates decreasing discharge time and voltage plateaus becoming less pronounced (Table 2). When the rate capability of the solid-state zinc-ion battery against the specific capacity in increasing cycles at various C-rates (Figure 7d), the specific capacity gradually decreased from 230 mAh g^–1^ at 0.9 C to 0.9 mAh g^–1^ at 211 C during the high-rate cycling, but upon returning to to 0.9 C, it recovered to 211 mAh g^–1^, indicating that ZrB_2_ exhibits excellent structural stability and reversibility. The Coulombic efficiency is nearly 87% at 1.4 C, indicating that the cell has a high degree of reversibility during charge–discharge processes.

**Table 2:** Capacitive Properties of ZrB_2_-Based Solid-State Hybrid Zinc-Ion Supercapacitor Obtained from GCD Curves (Figure 7c)

C	C_m_/mAh g^–1^	Coulombic Efficiency/%
0.9	230	73
1.4	142	87
2.1	95	91
5.3	38	97
19	11	99
55	3.6	100
211	0.9	100

**Figure 7:**
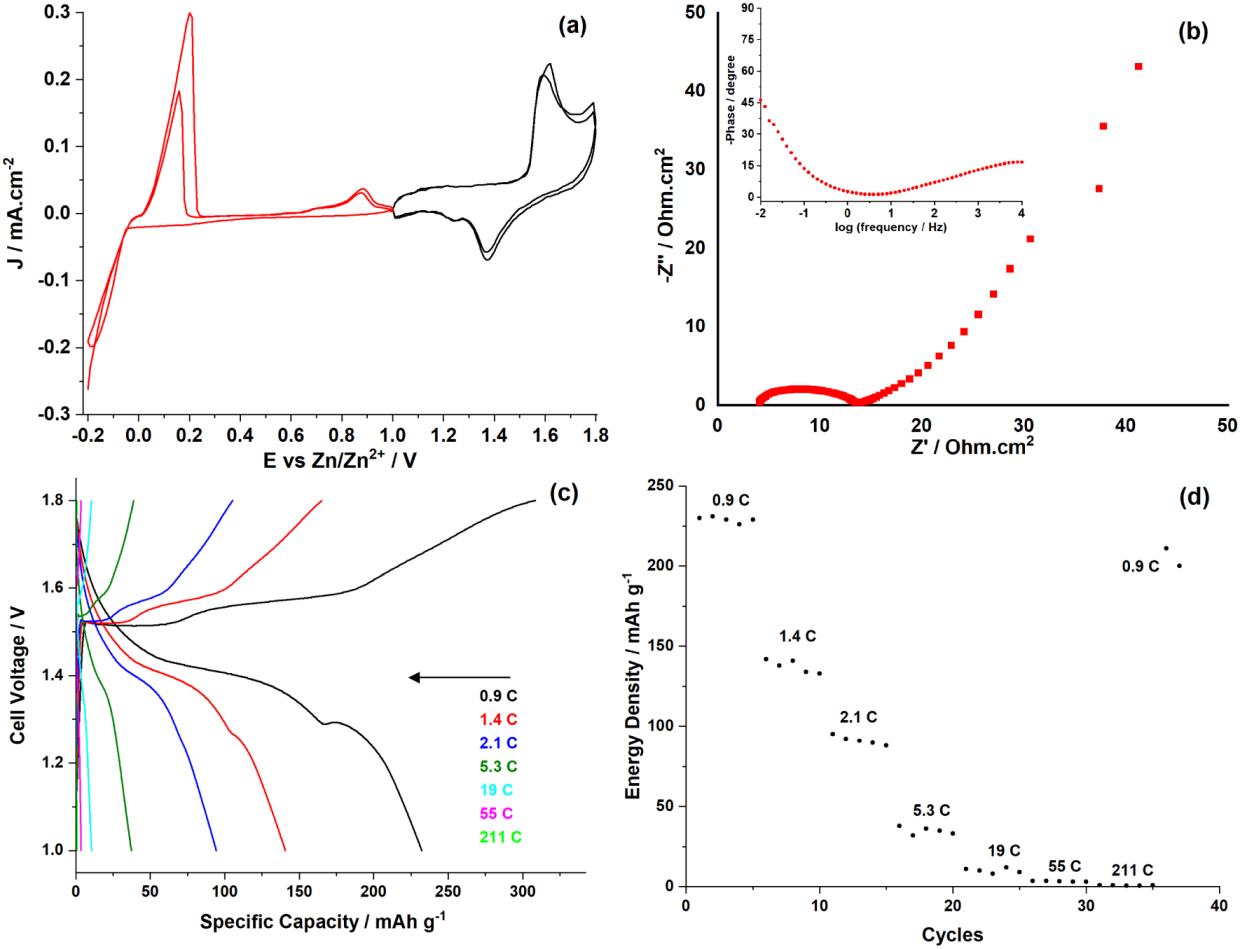
(a) CV curves of Zn anode and ZrB_2_ cathode at 0.5 mV s^–1^, (b) EIS curves, (c) charge–discharge curves recorded at 0.9 C–211 C rates, and (d) rate capability data for the solid-state zinc-ion battery cell and PVA//ZnSO_4_/MnSO_4_ electrolyte.

Table 3 compares the electrochemical performance of the ZrB_2_-based solid-state symmetric supercapacitors reported in this study with that of metal borides and their composites reported in the literature. One of the key strengths of this study is the successful integration of gel-derived ZrB_2_ into an all-solid-state symmetric configuration without requiring complex composite formation or mechanical activation processes. The citrate-gel approach enables uniform particle morphology and clean surface chemistry, facilitating direct electrode fabrication with conventional binders while maintaining an excellent interfacial contact with the PVA/Na_2_SO4 gel electrolyte. Furthermore, the demonstrated hybrid charge-storage mechanism (pseudocapacitive at low scan rates and EDLC-dominant at high scan rates) provides both high energy storage capability and rapid power delivery, making it a promising candidate for practical energy storage. Table 3 also presents a comparative evaluation of the solid-state zinc-ion battery configuration, demonstrating that our solid-state system exhibits higher capacities within a 1.8 V voltage window.

**Table 3:** A Comparison of Capacitive Performances for Supercapacitor Cells in Two-Electrode Systems with Metal Borides and Their Composites Given in the Literature with This Study

Electrode	Synthesis Method	Electrolyte	Cell Voltage/V	C	E/Wh kg^–1^	P/W kg^–1^	Cycle Life/% (cycles)	Ref.
ZrB_2_	Mechanical Activation	6 M KOH	1	1.862 mAh/g	4.2	150	79.1 (50)	^34^
ZrB_2_–SiC	Mechanical Activation	6 M KOH	1	0.327 mAh/g	0.4	75	69.3 (50)	^34^
ZrB_2_–ZrC	Mechanical Activation	6 M KOH	0.8	0.0021 mAh/g	6.6	149	99.8 (5000)	^35^
HfB_2_	Mechanical Activation	1 M Na_2_SO_4_	2	-	0.014	95.23	>98.7 (5000)	^31^
HfB_2_–SiC	Mechanical Activation	1 M Na_2_SO_4_	2	-	0.030	84.07	>98.7 (5000)	^31^
NiB/RGO	Reduction Method	6 M KOH	1.4	1073.4 F/g	22.1	724.9	72.4 (2500)	^60^
MoB/AC	Low-Temperature Synthesis	1 M H_2_SO_4_	1.4	-	14	16.8	80 (2000)	^61^
Mo–V–B_2_/C	Hydrothermal Process	1 M H_2_SO_4_	1	512 F/g	65.1	471.7	91.2 (10000)	^62^
Ni–Co–B/AC	Mircoimpinging Stream Reaction	2 M KOH	1.5	2415 F/g	74.3	18076	142 (5000)	^23^
FeB	Dipping-Drying and Chemical Synthesis	1 M Na_2_SO_4_	0.8	203.3 F/g	-	-	120 (3000)	^63^
LaB_6_	Chemical Vapor Deposition	1 M Na_2_SO_4_	1	17.3 mF/cm^2^	-	-	-	^64^
TiB_2_/MnO_2_	-	%30 KOH	1.2	1600 mAh/g	-	-	-	^65^
VB_2_/MnO_2_	-	%30 KOH	1.2	3100 mAh/g	-	-	-	^65^
CoB_0.5_	Mechanochemical Synthesis	6 M KOH	1	245 mAh/g	-	-	-	^66^
MoB_0.5_	Mechanochemical Synthesis	6 M KOH	1	1388 mAh/g	-	-	-	^66^
VB_0.5_	Mechanochemical Synthesis	6 M KOH	1	2158 mAh/g	-	-	-	^66^
VB_2_/MnO_2_	-	6 M KOH	0.9	2847 mAh/g	-	-	-	^67^
MoB_2_/MnO_2_	-	6 M KOH	0.7	1953 mAh/g	-	-	-	^67^
TiB_2_/MnO_2_	-	6 M KOH	0.9	973 mAh/g	-	-	-	^67^
MgB_2_/MnO_2_	-	6 M KOH	0.8	1183 mAh/g	-	-	-	^67^
TiB_2_/MnO_2_	-	ZnSO_4_/LiOH	1.8	148 mAh/g	-	-	55 (40)	^38^
MnO_2_	-	ZnSO_4_/LiOH	1.8	220 mAh/g	-	-	55 (40)	^38^
ZrB_2_	Gel Method	ZnSO_4_/MnSO_4_	1.8	230 mAh/g	-	-	-	**This study**
ZrB_2_	Gel Method	Na_2_SO_4_	1.4	-	4.4	11200	90.7	**This study**

## 4 Conclusion

The powders synthesized via the citric acid-based gel method predominantly consisted of ZrB_2_ with minor amounts of ZrO_2_ and exhibited a hexagonal-like shape. Electrochemical characterization of the ZrB_2_-based electrodes in 1 M Na_2_SO_4_ revealed a hybrid charge-storage mechanism. Dunn’s analysis showed that pseudocapacitive processes dominate at low scan rates (80%), while electric double-layer capacitance dominates at high scan rates (≈70%). This synergistic behavior ensures high energy storage at low rates and rapid power delivery at fast rates. In two-electrode solid-state symmetric cells with PVA/Na_2_SO_4_ gel electrolyte, the cell achieved a wide operating voltage of 1.4 V and maintained nearly rectangular CV profiles across 5–500 mV s^–1^. GCD tests yielded 6.5 Wh kg^–1^ at 0.25 mA cm^–2^ and 11200 W kg^–1^ at 16 mA cm^–2^, with Coulombic efficiencies rising from 52% to 83% as current density increased. The device exhibited low internal resistance (2.79 Ω cm^2^) and good cycling stability, retaining 90.7% of its initial capacitance after 5,000 cycles at 8 mA cm^–2^. Gel-derived ZrB_2_ is a promising electrode material for all-solid-state symmetric supercapacitors. By electrodepositing MnO_2_ (∼4.5 μg cm^–2^) at 1.8 V for 1 h onto the ZrB_2_ cathode, the zinc-ion cell achieves high specific capacity (230 mAh g^–1^ at 0.9 C), excellent rate capability up to 211 C, and 87% Coulombic efficiency at 1.4 C, confirming that gel-synthesized ZrB_2_ serves as an effective conductive framework and pseudocapacitive scaffold for multifunctional solid-state energy storage devices. The facile synthesis, structural characteristics, and hybrid energy-storage behavior yield cells with a wide cell potential, high electrochemical performance, and excellent cycle life. Future studies may focus on incorporating nanostructured carbons or conducting polymers with ZrB_2_ to develop advanced composites to further enhance capacity and rate performance, facilitating the development of high-power energy-storage applications.

## Data Availability

Data used is available throughout the manuscript text.
